# Factors Associated With Outcomes in Endoscopic Submucosal Dissection of Gastric Cardia Tumors: A Retrospective Observational Study

**DOI:** 10.1097/MD.0000000000001201

**Published:** 2015-08-07

**Authors:** Yae Su Jang, Bong Eun Lee, Gwang Ha Kim, Do Youn Park, Hye Kyung Jeon, Dong Hoon Baek, Dong Uk Kim, Geun Am Song

**Affiliations:** From the Department of Internal Medicine, Pusan National University School of Medicine, and Biomedical Research Institute, Pusan National University Hospital (YSJ, BEL, GHK, HKJ, DHB, DUK, GAS) and Department of Pathology, Pusan National University School of Medicine, Busan, Republic of Korea (DYP).

## Abstract

Tumors of the gastric cardia are among the most technically difficult lesions to remove by endoscopic submucosal dissection (ESD). This study aimed to evaluate the therapeutic outcomes of ESD in gastric cardia tumors according to clinicopathologic characteristics, and to assess the predictive factors for incomplete resection.

We conducted a retrospective observational study of 82 patients with adenomas and early cancers of the gastric cardia who underwent ESD between January 2006 and December 2013 at the Pusan National University Hospital. Therapeutic outcomes of ESD and procedure-related complications were analyzed.

En bloc resection, complete resection, and curative resection rates were 87%, 79%, and 66%, respectively. Deep submucosal invasion was the most common cause of noncurative resection in the cases in which complete resection was achieved. On multivariate analyses, hemispheric distribution (anterior hemisphere; odds ratio [OR] 4.808) and depth of tumor invasion (submucosal cancer; OR 22.056) were independent factors associated with incomplete resection. The rates of procedure-related bleeding, perforation, and stenosis were 6%, 1%, and 0%, respectively; none of the complications required surgical intervention.

In conclusion, ESD is a safe, effective, and feasible treatment for gastric cardia tumors. However, the complete resection rate decreases for tumors that are located in the anterior hemisphere or have deep submucosal invasion.

## INTRODUCTION

Endoscopic submucosal dissection (ESD) is a widely accepted treatment for premalignant lesions and early cancers of the stomach. The use of ESD has increased the rate of successful en bloc resection, and has made en bloc resection possible for tumors in difficult locations, such as the pylorus and cardia.^1–4^ However, ESD for tumors in difficult locations remains a technical challenge, with a low rate of successful resection, a long procedure time, and a high rate of complications compared with ESD for tumors in more favorable locations.^5–7^ The gastric cardia is a particularly constricted region located at the most proximal part of the stomach. This location makes a precise preoperative diagnosis and endoscopic resection of theses lesions challenging because of the sharp angle and narrow lumen. Consequently, surgery is often performed for gastric cardia tumors. However, surgical resection involves total or proximal gastrectomy, and may significantly degrade the patient's postoperative quality of life.^8^ Although ESD requires skillful endoscopic technique, it not only averts surgical risk but also improves the patient's quality of life by preserving the gastrointestinal tract.

Few studies have been published on the exact prevalence of gastric cardia tumors because of their rarity and a lack of a standard definition for gastric cardia tumors. A recent study on ESD for early gastric cancers (EGCs) reported that 2% were located at the gastric cardia.^9^ At our institution, gastric cardia tumors account for 2.9% of all gastric adenomas and EGCs (unpublished data). The number of ESD procedures performed for the treatment of gastric cardia tumors has increased with improvements in ESD techniques and devices, but published reports are scarce. Several studies have reported the results of ESD in the treatment of gastric cardia tumors as part of esophagogastric junction tumors,^2,10–13^ but there have been no studies regarding clinical outcomes on the basis of the clinicopathologic characteristics of gastric cardia tumors. Therefore, we aimed to evaluate the therapeutic outcomes of ESD in gastric cardia tumors, and to assess the possible predictive factors for incomplete resection.

## PATIENTS AND METHODS

### Patients

From January 2006 to December 2013, 2904 patients with early gastric tumors (adenomas and EGCs) were treated with ESD at Pusan National University Hospital (Busan, Korea). Of those, the records of 83 patients with 83 gastric cardia tumors were reviewed in this study. The inclusion criteria were a tumor of the gastric cardia, a tumor with an endoscopic morphology characteristic of a superficial neoplastic lesion as described by the Paris endoscopic classification,^14^ and a biopsy before the procedure interpreted as adenoma (low and high grade dysplasia) or adenocarcinoma. The exclusion criteria for entry into this study were the presence of severe systemic disease or advanced chronic liver disease and a history of gastric surgery. Of the 83 patients, 1 patient who previously underwent gastric surgery was excluded. Ultimately, a total of 82 patients with 82 gastric cardia tumors were included in this study. All patients with EGC underwent abdominal computerized tomography (CT) before ESD to determine the presence of lymph node or distant metastases. Additionally, endoscopic ultrasonography (EUS) was performed to rule out submucosal invasion in most EGC cases. All patients agreed to undergo ESD after explanation of the risks and benefits, including complications of ESD and the possible necessity for additional surgical treatment. Written informed consent was obtained from all patients before ESD, and the study protocol was reviewed and approved by the Institutional Review Board of Pusan National University Hospital (E-2014141).

### Assessment of Tumor Location and Directional Distribution

Gastric cardia tumors were defined as tumors of which the center was located within 2 cm distal to the esophagogastric junction.^15^ The esophagogastric junction was defined as the oral-side end of the fold that is present continuously from the gastric lumen,^16^ as well as the anal-side end of the fine, parallel, uniformly distributed longitudinal vessels in the lower part of the esophagus.^17^

**FIGURE 1:**
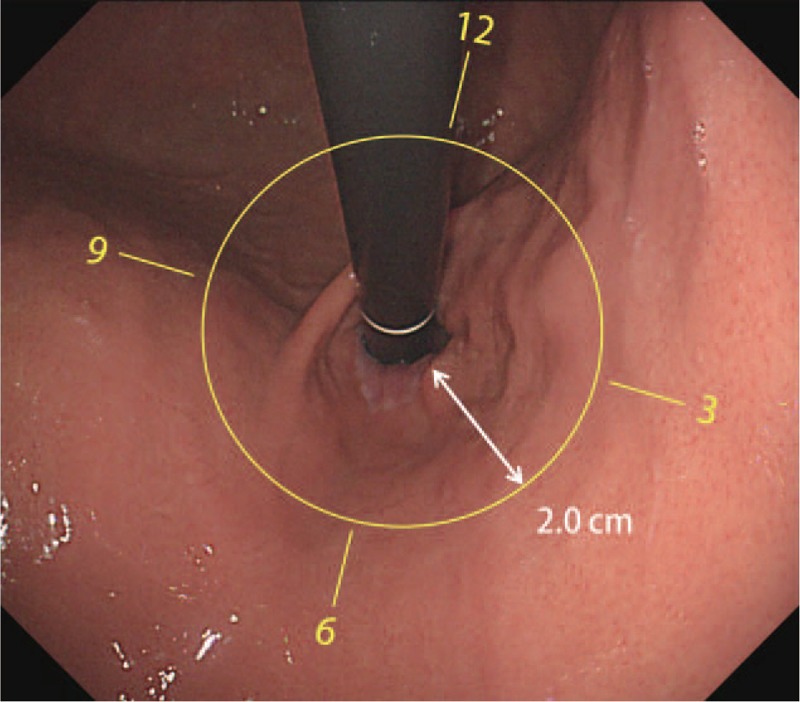
Endoscopic assessment of gastric cardia tumors. Gastric cardia tumors are defined as tumors of which center is located within 2 cm distal to the esophagogastric junction. A clock-face orientation in the retroflexed position (with the lesser curve [LC] of the stomach in contiguity with the 6 o’clock position of the cardia) is used to classify directional distribution into 4 quadrants.

The location of tumors was classified according to their esophageal extension above the esophagogastric junction. Cardia only type (C-type) tumors were confined to the gastric side below the esophagogastric junction and cardia-esophagus type (CE-type) tumors extended to the lower esophagus beyond the esophagogastric junction. In the retroflexed position, a clock-face orientation of the endoscope (with the lesser curve of the stomach in contiguity with the 6 o’clock position of the cardia) was used to characterize the directional distribution according to quadrant: first quadrant (12–3 o’clock), second quadrant (3–6 o’clock), third quadrant (6–9 o’clock), and fourth quadrant (9–12 o’clock) (Figure 1). The lesions were also classified according to anterior and posterior hemispheric distribution: anterior hemisphere from 6 to 12 o’clock and posterior hemisphere from 12 to 6 o’clock. When a lesion spanned 2 or more quadrants, the central portion of the lesion was used to designate its predominant location.

The macroscopic shapes of lesions were categorized as either protruding (I), nonprotruding and nonexcavated (II), or excavated (III). Type II lesions were subclassified as slightly elevated (IIa), ﬂat (IIb), or slightly depressed (IIc). All lesions were also classified into 3 groups: elevated (I, IIa), flat (IIb), and depressed (IIc, III) types.

Hiatal hernia was defined as a circular extension of the gastric mucosa above the diaphragmatic hiatus >2 cm in axial length.^18^

### ESD Procedures

ESD procedures were performed by 2 experienced endoscopists (GHK and GAS), using a single-channel endoscope (GIF-H260 or GIF-Q260; Olympus Co., Ltd., Tokyo, Japan). Procedures were performed with the patient under conscious sedation with cardio-respiratory monitoring. For sedation, midazolam 5 to 10 mg and meperidine 25 mg were administered intravenously. Propofol was administered as needed during the procedure. First, argon plasma coagulation was used to mark the borders of the lesion, which had been identified by conventional endoscopy or chromoendoscopy with application of an indigo carmine solution. After marking, a saline solution (0.9% saline with a small amount of epinephrine and indigo carmine) was injected submucosally around the lesion in order to elevate it off the muscular layer.

For C-type tumors, a circumferential mucosal incision was made outside the marking dots with an IT knife (Olympus) and/or a Flex knife (Olympus) in the retroflexed position. Next, submucosal dissection was performed, using the knife to completely remove the lesion. If necessary during the procedure, the submucosal injection was repeated and endoscopic hemostasis was achieved. A high-frequency electrosurgical current generator (Erbotom VIO 300D; ERBE, Tübingen, Germany) was used during marking, mucosal incision, submucosal dissection, and hemostasis.

**FIGURE 2:**
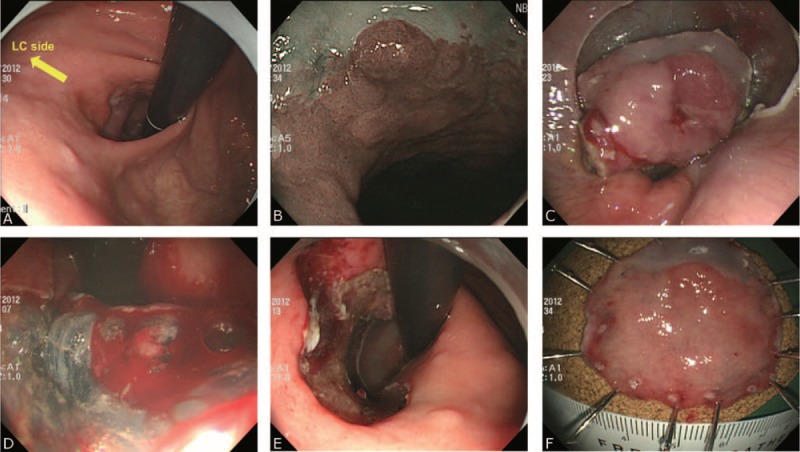
Example of endoscopic submucosal dissection for a gastric cardia tumor with esophageal extension. (A) A slightly elevated lesion is seen in the 3 to 6 o’clock quadrant of the cardia with the lesser curve (LC) of the stomach in contiguity with the 6 o’clock position of the cardia. (B) Extension of the tumor to the lower esophagus beyond the esophagogastric junction is clearly seen on narrow band imaging. (C) Mucosal incision and submucosal dissection are started from the lower esophagus in the forward position. (D) Submucosal dissection is continued in the gastric side in the retroflexed position. (E) The lesion is completely removed. (F) The resected specimen.

For CE-type tumors, the mucosal incision and dissection were started at the lower esophagus in the forward position. The remainder of the ESD of CE-type tumors was then the same as that for C-type tumors (Figure 2). If mucosal incision of the proximal part of the lesion was impossible, submucosal dissection was started from the distal part.

### Histopathological Evaluation

Resected specimens were fixed in formalin and serially sectioned at 2-mm intervals in order to assess tumor involvement in the lateral and vertical margins. The tumor size, depth of invasion, presence of ulceration, degree of differentiation, and lymphovascular invasion (LVI) were evaluated microscopically according to the Japanese classification of gastric carcinoma.^19^

### Outcome Parameters

The primary outcome parameter was the success of the endoscopic resection such as the rates of en bloc resection, complete resection, and curative resection. The secondary outcome parameters were procedure time, procedure-related complications, and local recurrence rate. En bloc resection was defined as a resection in a single piece. Complete resection was defined as successful en block resection, with lateral and vertical margins histologically free of neoplasm. Curative resection was defined as a complete resection that fulfilled the following criteria:^13,19,20^ mucosal cancer, differentiated-type adenocarcinoma, no LVI, without ulceration, irrespective of tumor size, mucosal cancer, differentiated-type adenocarcinoma, no LVI, with ulceration, tumor size ≤3 cm, minute submucosal cancer invasion ≤500 μm, differentiated-type adenocarcinoma, no LVI, tumor size ≤3 cm, or mucosal cancer, undifferentiated-type adenocarcinoma, no LVI, without ulceration, tumor size ≤2 cm.

Procedure time was defined as the time from the start of the marking to the complete removal of the tumor. Procedure-related bleeding was defined as bleeding proven by endoscopic evaluation within 24 h, clinical evidence of melena or hematemesis, or massive bleeding requiring transfusion.^4^ Bleeding occurring during the ESD procedure that was treated endoscopically was not regarded as procedure-related bleeding. Perforation was endoscopically diagnosed during the procedure or by the presence of free air on plain chest radiography after ESD. Procedure-related stenosis was diagnosed with endoscopy and defined as present when a standard 10-mm diameter endoscope could not be passed through the esophagogastric junction.

### Follow-Up

All patients who were treated with ESD underwent postprocedural chest and abdominal radiography and second-look endoscopy on the following day to detect any perforation or bleeding. Proton pump inhibitors and sucralfate were administered to relieve pain, prevent procedure-related bleeding, and promote ulcer healing. Patients without serious symptoms or adverse events were permitted to start food intake the day after the procedure and were discharged within 3 to 4 days.

In cases of curative resection, follow-up endoscopy was conducted 6 months after ESD and annually thereafter. In EGC cases with curative resection, abdominal CT, chest radiography, and laboratory measurements of tumor markers were performed 6 months after ESD and annually thereafter. In EGC cases with noncurative resection such as those with LVI, a positive vertical margin, or deep submucosal invasion, an additional gastrectomy with lymph node dissection was recommended to all patients for curative resection. However, for patients who refused a surgical operation, follow-up endoscopy with biopsies and abdominal CT were conducted 1 to 2 months and 4 to 6 months after ESD.

### Statistical Analysis

Variables were expressed as medians or interquartile ranges (IQR) and simple proportions. Statistical significance was evaluated by use of the Mann–Whitney *U* test or Kruskal–Wallis test for continuous variables, and the χ^2^ test or Fisher's exact test for categorical variables. Factors associated with incomplete resection were assessed by use of logistic regression analysis. Univariate comparisons were expressed as odds ratios (OR) with 95% confidence intervals (CI). Significant factors on univariate analyses, defined as a *P* < 0.05, or factors with clinical correlation were included in the multivariate model to assess for independent factors for incomplete resection. A *P* value of <0.05 was considered statistically significant. The statistical calculations were performed with SPSS version 21.0 for Windows software (SPSS, Inc., Chicago, IL).

## RESULTS

### Clinicopathologic Characteristics of Patients and Gastric Cardia Tumors

Clinicopathologic characteristics of the patients and tumors are summarized in Table 1. The patients included 71 males and 11 females with a median age of 68 years (range, 43–84 years). At the index endoscopy, 66 (80%) lesions were confined to the gastric cardia, and 16 (20%) extended to the lower esophagus. The directional distributions were 12 to 3 o’clock quadrant in 7 lesions, 3 to 6 o’clock quadrant in 30, 6 to 9 o’clock quadrant in 39, and 9 to 12 o’clock quadrant in 6. Therefore, the hemispheric distributions were anterior in 45 (55%), and posterior in 37 (45%). The tumor sizes were ≤10 mm in 25 lesions (30%), 10 to 20 mm in 39 (48%), and >20 mm in 18 (22%). The pathologic diagnoses of the lesions were 33 adenomas (40%) and 49 cancers (60%) (differentiated-to-undifferentiated-type adenocarcinoma, 47:2).

**TABLE 1:**
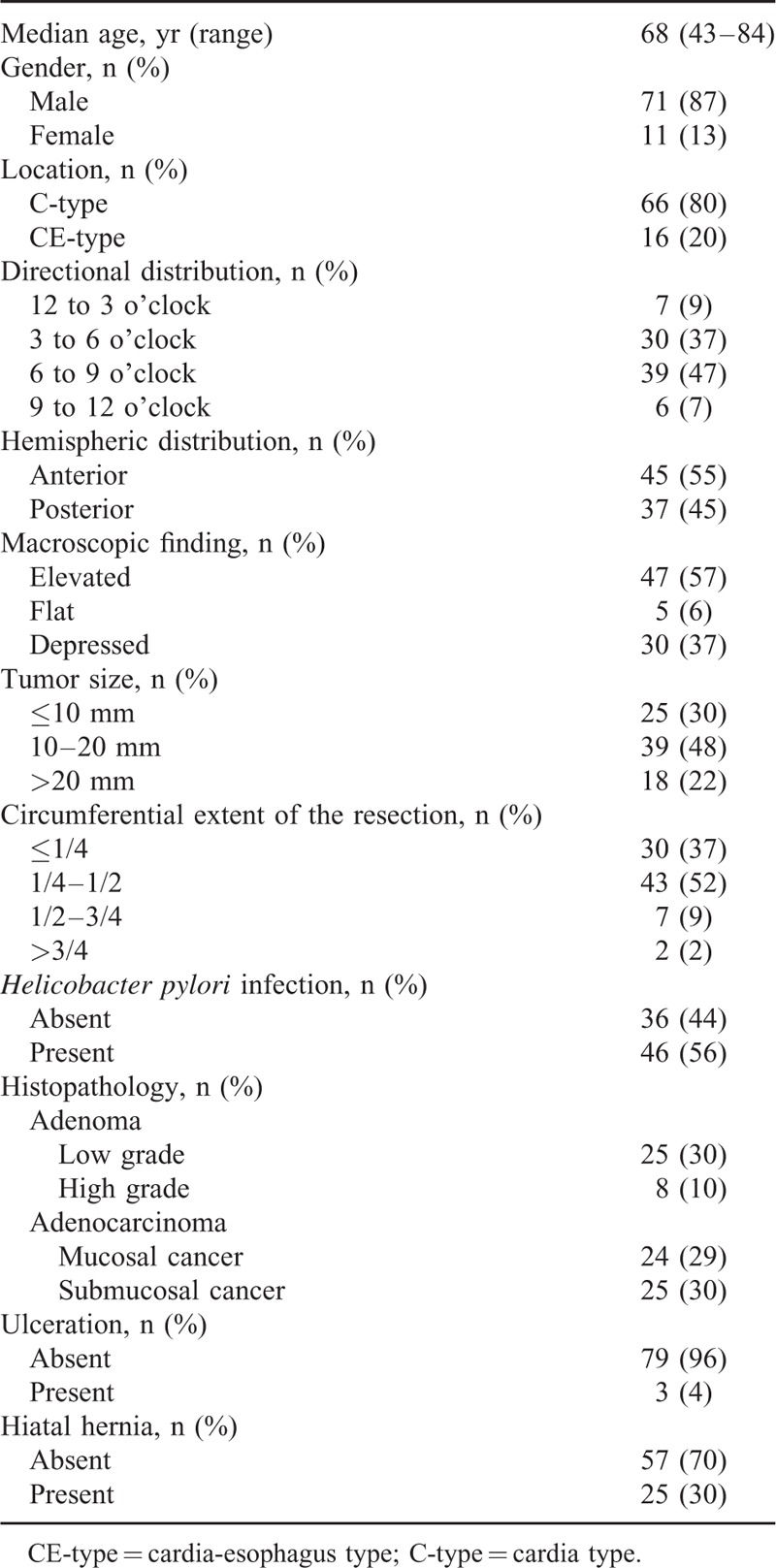
Clinicopathologic Characteristics of Patients With Gastric Cardia Tumors

### ESD Outcomes

Table 2 shows the outcomes for ESD of gastric cardia neoplasms. The en bloc resection rate was 87% (71/82) and the piecemeal resection rate was 13% (11/82). Of the en bloc resected lesions, 6 had a positive margin (lateral involvement with the tumor cells in 5 and vertical involvement with tumor cells in 1). Therefore, the complete resection rate was 79% (65/82). Of 36 completely resected EGCs, deep submucosal invasion (>500 μm from the muscularis mucosa) was found in 10 cases and LVI was found in 1 case with mucosal cancer. As a result, the curative resection rate was 66% (54/82). The median procedure time was 37 min (IQR, 25.0–58.5).

**TABLE 2:**
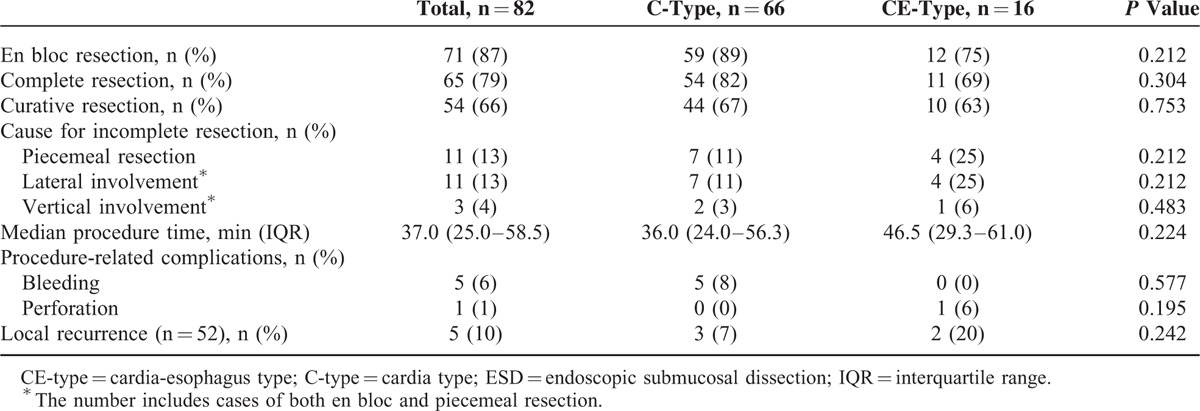
Therapeutic Outcomes of ESD for Gastric Cardia Tumors According to Tumor Location

Tables 2 and 3 show the ESD outcomes according to location and clock-face orientation. The en bloc resection and complete resection rates in C-type and CE-type tumors were 89% and 75%, and 82% and 69%, respectively. There were no significant differences in the en bloc resection and complete resection rates between the 2 types (*P* = 0.212 and *P* = 0.304, respectively). The median procedure time in C-type and CE-type tumors was 36 and 47 min, respectively (*P* = 0.224). The complete resection rate varied according to the clock-face directions; the complete resection rate was lowest in the 6 to 9 o’clock quadrant (69%), which was not statistically significant (*P* = 0.092).

**TABLE 3:**
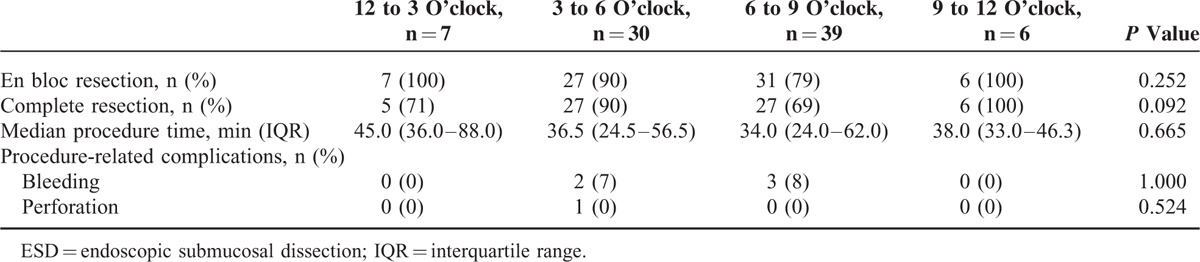
Therapeutic Outcomes of ESD for Gastric Cardia Tumors According to Clock-Face Direction

There were significant differences in the complete resection rates in relation to histopathology (adenoma vs mucosal cancer vs submucosal cancer, 88% vs 88% vs 60%, respectively; *P* = 0.028) (Table 4). The complete resection rate decreased in lesions located in the anterior hemisphere compared to those located in the posterior hemisphere, though this did not reach statistical significance (73% vs 86%, *P* = 0.144). The complete resection rate decreased in lesions with tumor size >20 mm and increased in the presence of hiatal hernia, but these did not reach statistical significance (*P* = 0.187 and *P* = 0.196, respectively).

**TABLE 4:**
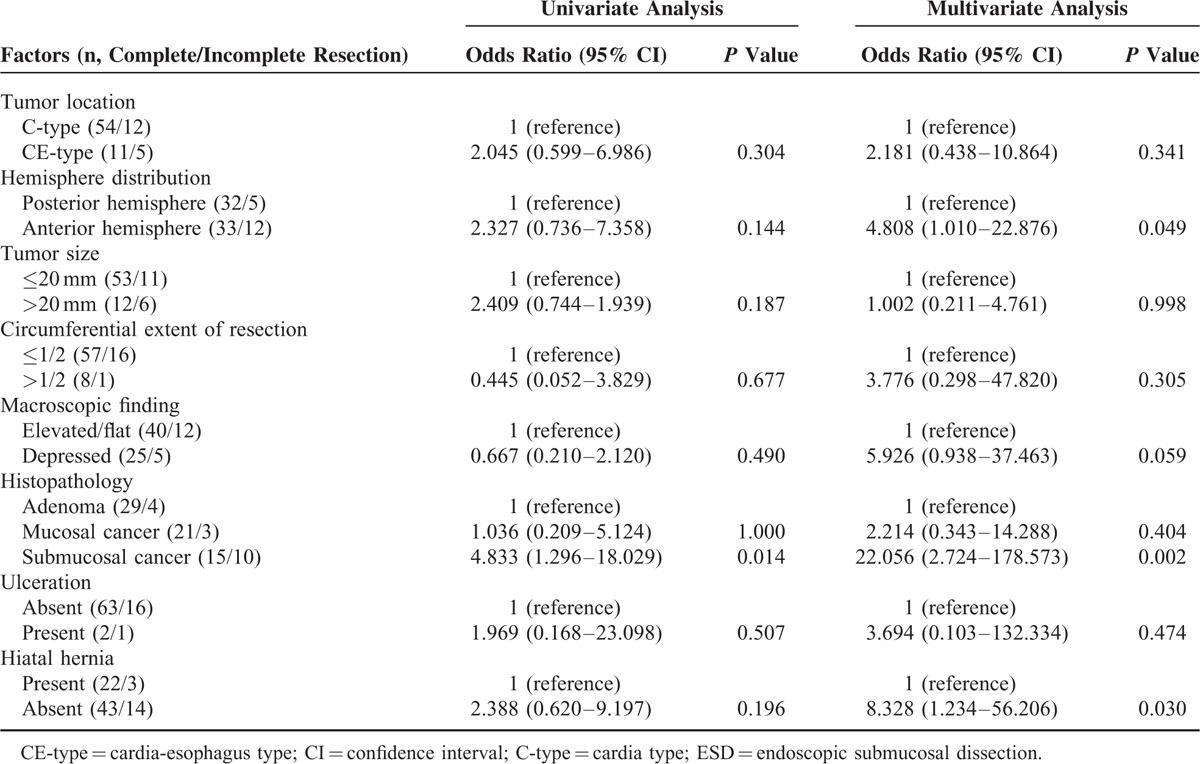
Univariate and Multivariate Analyses for Incomplete Resection With ESD for Gastric Cardia Tumors

### Multivariate Analysis for Factors Influencing Incomplete Resection

Multivariate logistic regression analyses revealed hemisphere distribution, histopathology, and hiatal hernia to be relevant independent factors influencing incomplete resection (Table 4). Incomplete resection rates increased in lesions located in the anterior hemisphere (OR 4.808, 95% CI 1.010–22.876; *P* = 0.049) and in lesions with submucosal cancer (OR 22.056, 95% CI 2.724–178.575; *P* = 0.002). With regard to the hiatal hernia, the absence of a hiatal hernia was independently associated with incomplete resection (OR 8.328, 95% CI 1.234–56.206; *P* = 0.030). However, tumor size, circumferential extent of resection, macroscopic findings, and ulceration were not related to incomplete resection.

### Complications

The rates of procedure-related bleeding and perforation were 6% and 1%, respectively (Table 2). Procedure-related bleeding was observed in 5 cases (bleeding occurred in 2 cases on the 14th day after ESD), but all bleeding was managed successfully with endoscopic hemostasis. All cases of bleeding occurred in C-type lesions, but the difference in bleeding rates between C-type and CE-type tumors was not statistically significant (*P* = 0.577). Procedure-related perforation was encountered in 1 patient with a CE-type tumor, and was detected only on the chest X-ray after the ESD procedure. The patient was treated nonoperatively with antibiotics and restricted oral intake. Procedure-related stenosis was not encountered in any cases, including 2 cases in which the circumferential extent of the resection was more than 3/4.

### Operation and Local Recurrence

Of 24 noncurative EGC lesions, 20 were deep submucosal cancers and 4 were mucosal cancers. We recommended additional surgical resection for all 20 patients with deep submucosal cancer. Six of these patients underwent surgical resection, but 8 patients did not undergo additional surgery because of advanced age, poor performance status, or refusal to undergo further surgery. The other 6 patients with deep submucosal cancer were lost to follow-up. Of the 4 patients with noncurative mucosal cancer, 1 patient underwent surgical resection because of the presence of LVI. The other 3 patients continued with follow-up. There was no mortality related to ESD or subsequent surgery.

**FIGURE 3:**
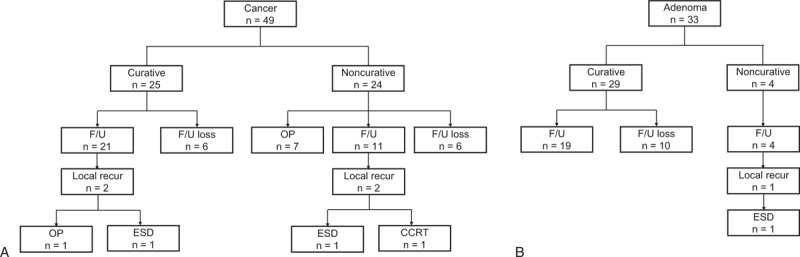
Outcomes of patients who underwent endoscopic submucosal dissection for gastric cardia tumor (A, B). CCRT = concurrent chemoradiotherapy; ESD = endoscopic submucosal dissection; F/U = follow-up; OP = operation.

Fifty-two of the 82 patients treated with ESD were followed up for ≥6 months (Figure 3). During the median follow-up period of 13 months (IQR 6−60 months), 3 deaths occurred due to cholanigiocarcinoma, gallbladder cancer, and liver cirrhosis. Recurrences occurred in 5 cases, and recurrent lesions were found in 3 of 11 incompletely resected cases (6, 9, and 45 months after ESD) and in 2 of 41 completely resected cases (12 and 28 months after ESD). Three recurrent cases were treated by a second ESD, and there have been no additional recurrences in these cases. One of the other 2 recurrent cases was treated by operation and the other by concurrent chemoradiotherapy.

## DISCUSSION

Generally, difficulties in performing ESD are the result of several clinicopathologic factors, such as the location, size, and depth of tumor invasion, and the complete resection rate and procedure time are usually proportional to the technical difficulty.^5,6,21^ Gastric cardia tumors are among the most difficult lesions to remove with ESD. In the present study, we showed that the technical outcomes of ESD in gastric cardia tumors were influenced significantly by hemispheric distribution, depth of tumor invasion, and hiatal hernia. These results provide important information to endoscopists, especially novices and trainees, for assessing the potential difficulties in performing ESD for gastric cardia tumors before undertaking the procedure.

Because the lumen of the lower esophagus is narrow and its thin wall constantly moves with respiration and cardiac contractions, the manipulation of endoscopic knives, especially the IT knife, is difficult. Therefore, we assumed that the complete resection rate in CE-type lesions would be lower than that in C-type lesions. However, with respect to the location of gastric cardia tumors, there was no difference in the complete resection rate between CE-type and C-type lesions (69% vs 82%). This observation may be explained as follows. The 2 endoscopists who performed ESD in the present study had extensive experience in ESD, and performed all ESD procedures in the present study. If less experienced endoscopists had been included in this study, the complete resection rate of the CE-type lesions may possibly have been lower than that in the C-type lesions. In addition, CE-type lesions accounted for only 20% of our cases, and if more CE-type lesions had been included in our study, our results might have been different.

Our classification of gastric cardia tumors according to hemispheric distribution revealed that the complete resection rate for lesions located in the anterior hemisphere (73%) was lower than that for lesions located in the posterior hemisphere (86%). On multivariate analyses, the incomplete resection rate increased in lesions located in the anterior hemisphere compared to those located in the posterior hemisphere (OR 4.808, 95% CI 1.010–22.876). In our experience, the posterior wall of the gastric cardia is relatively flat, and it is less difficult to orient the knife parallel to the submucosal layer beneath the tumor. In contrast, because the anterior side of the gastric cardia is somewhat concave, it is more difficult to orient the knife parallel to the submucosal layer beneath the tumor. These differences could explain our results. When approaching anterior hemisphere lesions, additional techniques such as traction methods with a transnasal or double channel endoscope^22,23^ or the use of a multibending endoscope^24^ may assist in the performance of a successful ESD.

In the present study, the noncurative resection rate was significantly higher in submucosal cancers than in adenomas and mucosal cancers (OR 22.056, 95% CI 2.724–178.575). This is consistent with data from previous studies.^10,12^ With regard to the depth of tumor invasion, curative resection was defined when submucosal cancer invasion was ≤500 μm. However, it was difficult to accurately predict the depth of invasion of gastric cardia EGCs preoperatively in the present study. This could explain the higher noncurative resection rate in submucosal cancer cases.

Interestingly, the absence of a hiatal hernia was associated with incomplete resection in the present study (OR 8.328, 95% CI 1.234–56.206). Why does the complete resection rate increase in the presence of hiatal hernia? If there is no hiatal hernia in a patient with a gastric cardia lesion, the proximal portion of the lesion is located at the narrowest, funnel-like portion of the stomach. As a result, it is very difficult to precut and dissect the proximal portion of the lesion. On the other hand, if a hiatal hernia is present, the lesion is displaced into the tunnel-like hernia sac. This allows more working space for ESD of the lesion, especially the proximal portion of the lesion. This phenomenon might explain the higher complete resection rate in the presence of a hiatal hernia.

In the present study, the en bloc resection, complete resection, and curative resection rates for gastric cardia tumors were 87%, 79%, and 66%, respectively, which are consistent with data from previous studies.^11–13^ However, the complete resection and curative resection rates were lower than those for gastric tumors located in other regions.^25^ This is especially true for gastric cardia EGCs in which only 24 of 49 (49%) achieved curative resection in the present study. Even though EUS was performed to rule out submucosal invasion in most cases before ESD, the main cause of noncurative resection was deep submucosal invasion (20/24, 83%). When the tumor is located near the esophagogastric junction, it is difficult to position the ultrasound transducer optimally, with resultant pseudo-thickening and a poor visualization of the gastric wall layers.^26,27^ These limitations may have led to an underestimation of the depth of invasion as measured by EUS. Although the noncurative resection rate in the present study is somewhat higher than seen in previous studies,^2,10–13^ our findings showing that the incidence of submucosal cancer was high in the final histopathological results after ESD for gastric cardia tumors are consistent to those of previous studies.^2,11,13^ This suggests that gastric cardia EGCs have a high malignant potential, indicating the need for caution during ESD. However, since the depth of invasion is difficult to accurately diagnose preoperatively, even using EUS, detailed histopathological investigation of the ESD resected specimen is mandatory.

In the present study, procedure-related bleeding was seen in 5 cases (6%), and there was no difference in the bleeding rate between C-type and CE-type lesions. The risk factors for stenosis occurring after ESD for gastric cardia tumors are known to be a circumferential mucosal defect >3/4 or a longitudinal extent of >5 cm.^9^ In our study, a circumferential mucosal defect >3/4 was present in 2 cases, and a longitudinal extent >5 cm was present in 5 cases. However, procedure-related stenosis did not occur after ESD in any case.

Previous studies have shown that in patients who had undergone curative resection with ESD there was neither local recurrence nor distant metastases during follow-up.^11–13^ However, in the present study, local recurrences occurred during the long-term follow-up in 2 of 25 lesions, which had been resected curatively for gastric cardia EGC. One case occurred with mucosal cancer and the other occurred with minute submucosal cancer. Review of the pathologic specimens in these cases confirmed the curative nature of the ESD. However, in both cases, the cancer cells showed a multifocal pattern. This suggests that even though the cancer was completely resected in the pathologic specimen, there was still the possible presence of cancer cells outside the resected specimen in these EGC lesions with a multifocal pattern. Therefore, close follow-up is indicated in patients with a multifocal pattern even when curative resection has apparently been achieved.

To our knowledge, this is the first study to show the outcomes of ESD according to clinicopathologic characteristics and to evaluate the predictive factors for incomplete resection in gastric cardia tumors. Our study differs from previous studies in several ways. First, in those studies, the term “esophagogastric junction tumor” was used instead of gastric cardia tumor, and esophagogastric junction tumor was defined as a tumor located from 1 cm above to 2 cm below the esophagogastric junction on the basis of the Siewert classification system (type II).^28^ Esophagogastric junction tumors usually include lower esophageal tumors (ie, Barrett's tumors) as well as gastric tumors (true cardia tumors). Furthermore, it is inappropriate to apply the definition of curative resection in ESD for EGCs to Barrett's cancer. Therefore, in the present study, we strictly defined gastric cardia tumors as tumors whose center was located within 2 cm distal to the esophagogastric junction. In doing so, we tried to include only true gastric tumors in the present study. Second, we classified the lesions according to the presence of esophageal extension, and separately evaluated the results of ESD. Moreover, we showed the relationship between directional distribution of the tumor and outcomes with ESD using the clock-face orientation. We believe this provides informative data in assessing the potential difficulties in performing ESD in certain cases.

Nonetheless, this study has several limitations. First, the study is a single-center study and is subject to the biases inherent in retrospective observational studies. Although most results of ESD were prospectively collected after the endoscopists at the time of the endoscopy, the clock-face direction designations were retrospectively assigned by review of the endoscopic images.^4^ However, we routinely photograph gastric cardia lesions with the endoscope in the retroflexed position, with the lesser curvature of the stomach aligned in the 6 o’clock position. In addition, we assessed the pre-ESD, procedural, and post-ESD endoscopic images in careful detail. Therefore, we believe that any error from assigning the distribution of lesions would be small, and would have been unlikely to affect our results. Second, there were some technical differences between the 2 endoscopists in our study, including differences in the selection of knives, the time required to change equipment, and the amounts of injected materials. Finally, although we tried to include only true gastric cardia tumors using a strict definition, there is still a possibility that some Barrett's tumors might have been included because almost all Barrett's cancers occur in the ultrashort-segment or short-segment Barrett's epithelium in Korean patients.^29^

In conclusion, our results showed a high rate of complete resection and a low rate of procedure-related complications for ESD of gastric cardia tumors. Endoscopists considering ESD for a gastric cardia tumor should be aware that lesions in anterior hemispheric location or showing submucosal invasion have a decreased complete resection rate. ESD may be technically challenging, but can be an effective and safe therapy in the hands of endoscopists who have sufficient skill and knowledge in the treatment of gastric cardia tumors. However, the use of ESD should be carefully considered for gastric cardia EGCs with suspected mucosal invasion after pretreatment work-up because of their higher frequency of deep submucosal invasion. Additional prospective multicenter studies with a larger number of cases may provide additional information regarding the use of ESD for gastric cardia tumors.
